# Radiomics for diagnosis of dual-phenotype hepatocellular carcinoma using Gd-EOB-DTPA-enhanced MRI and patient prognosis

**DOI:** 10.1007/s00432-019-03062-3

**Published:** 2019-10-29

**Authors:** Xialing Huang, Liling Long, Jieqin Wei, Yajuan Li, Yuwei Xia, Panli Zuo, Xiangfei Chai

**Affiliations:** 1 Department of Radiology, The First Affiliated Hospital of Guangxi Medical University, Guangxi Medical University, No. 6 Shuangyong Road, Nanning, Guangxi China; 2 Department of Radiology, The First Affiliated Hospital of Guangxi Medical University, No. 6 Shuangyong Road, Nanning, Guangxi China; 3 Department of MRI, The First People’s Hospital of Nanning, The First People’s Hospital of Nanning, No.89 Qixing Road, Nanning, Guangxi China; 4 Huiying Medical Technology Co., Ltd, Room A206, B2, Dongsheng Science and Technology Park, HaiDian District, Beijing City, 100192 China

**Keywords:** Dual-phenotype hepatocellular carcinoma, Radiomics, Gd-EOB-DTPA-enhanced MRI, Prognosis

## Abstract

**Purpose:**

To describe the clinical characteristics and outcomes of patients with dual-phenotype hepatocellular carcinoma (DPHCC) and investigate the use of radiomics to establish an image-based signature for preoperative differential diagnosis.

**Methods:**

This study included 50 patients with a postoperative pathological diagnosis of DPHCC (observation group) and 50 patients with CK7- and CK19-negative HCC (control group) who attended our hospital between January 2015 and December 2018. All patients underwent Gd-EOB-DTPA-enhanced MRI within 1 month before surgery. Arterial phase (AP), portal venous phase (PVP), delayed phase (DP) and hepatobiliary phase (HBP) images were transferred into a radiomics platform. Volumes of interest covered the whole tumor. The dimensionality of the radiomics features were reduced using LASSO. Four classifiers, including multi-layer perceptron (MLP), support vector machines (SVM), logistic regression (LR) and K-nearest neighbor (KNN) were used to distinguish DPHCC from CK7- and CK19-negative HCC. Kaplan–Meier survival analysis was used to assess 1-year disease-free survival (DFS) and overall survival (OS) in the observation and control groups.

**Results:**

The best preoperative diagnostic power for DPHCC will likely be derived from a combination of different phases and classifiers. The sensitivity, specificity and accuracy of LR in PVP (0.740, 0.780, 0.766), DP (0.893, 0.700, 0.798), HBP (0.800, 0.720, 0.756) and MLP in PVP (0.880, 0.720, 0.798) were better performance. The 1-year DFS and OS of the patients in the observation group were 69% and 78%, respectively. The 1-year DFS and OS of the patients in the control group were 83% and 85%, respectively. Kaplan–Meier survival analysis showed no statistical difference in DFS and OS between groups (*P* = 0.231 and 0.326), but DFS and OS were numerically lower in patients with DPHCC.

**Conclusion:**

The radiomics features extracted from Gd-EOB-DTPA-enhanced MR images can be used to diagnose preoperative DPHCC. DPHCC is more likely to recur and cause death than HCC, suggesting that active postoperative management of patients with DPHCC is required.

## Background

Hepatocellular carcinoma (HCC) is among the most common malignancies and the third leading cause of cancer-related death worldwide (Torre et al. 2015). As a subtype of HCC, dual-phenotype hepatocellular carcinoma (DPHCC) is included in the evidence-based practice guidelines for pathological diagnosis of primary liver cancer in China (2015 update) (Chinese Society of Liver Cancer et al. 2015). Microscopically, the morphology of DPHCC is similar to HCC, but DPHCC simultaneously shows the expression of markers of HCC (hepatocyte-1, glypican-3) and cholangiocellular carcinoma (CK7, CK19) in immunohistochemistry.

A preliminary diagnosis of DPHCC on preoperative imaging is essential for clinical decision-making, as evidence suggests that CK19-positive HCC has high invasive, proliferative and migration abilities (Lee et al. 2012, 2013). Furthermore, HCC expressing a cholangiocyte phenotype is highly aggressive, and CK7 and CK19 are potential predictors of poor prognosis in patients with cholangiocarcinoma after surgery (Liu et al. 2018). Gadolinium-ethoxybenzyl-diethylenetriamine pentaacetic acid (Gd-EOB-DTPA)-enhanced magnetic resonance imaging (MRI) has an important role in the diagnosis of HCC (Zhao et al. 2018; Esterson et al. 2015). However, DPHCC is similar to the cellular components of conventional HCC in pathology, so the imaging and clinical features of DPHCC and HCC are similar, making differential diagnosis on preoperative imaging challenging. In 2012, Lambin et al. introduced radiomics (Lambin et al. 2012). Radiomics has been successfully applied in the screening, diagnosis, treatment, and evaluation of multiple tumor types (Huang et al. 2018; Lee et al. 2019). Oyama et al. applied radiomics of non-contrast-enhanced three-dimensional T1-weighted MR images to classify hepatic tumors (Oyama et al. 2019). Gao et al. performed preoperative pathological grading prediction of hepatocellular carcinoma based on MRI radiomics (Gao et al. 2019). The objective of this study was to describe the clinical characteristics and outcomes of patients with DPHCC and investigate the use of radiomics to establish an image-based signature for use in preoperative differential diagnosis.

## Materials and methods

### Clinical data

Inclusion criteria: patients with a postoperative pathological diagnosis of DPHCC (observation group) and patients with CK7- and CK19-negative HCC (control group) who attended our hospital between January 2015 and December 2018. Patients underwent Gd-EOB-DTPA-enhanced MRI within 1 month before surgery. Exclusion criteria: patients who underwent interventional therapy before surgery. Patients who pathologically confirmed combined hepatocellular carcinoma and cholangiocarcinoma (cHCC-CC) and intrahepatic cholangiocellular carcinoma (ICC).

### Image acquisition

All patients underwent plain and enhanced MR imaging (10 mL Gd-EOB-DTPA at 0.25 mmol/mL, Germany Bayer Healthcare Co.) using a Siemens Verio 3.0 T MRI scanner with a 12-channel body phased-array coil. Gd-EOB-DTPA was administered as a bolus injected at a rate of 2 mL/s through the cubital vein followed by a 20 mL saline chaser administered at the same rate. The scanning parameters of T1WI volume interpolated body examination (VIBE) were repetition time (TR) 3.9 ms, echo time (TE) 1.4 ms, flip angle 15°, field of view (FOV) 350 mm, matrix 168 × 320, voxel size 1.6 × 1.1 × 4.5 mm, signal to noise ratio (SNR) 1.00, 4.5 mm section thickness. The scanning parameters of T2WI using BLADE technique were TR 2930 ms, TE 189 ms, FOV 400 mm, voxel size 1.3 × 1.3 × 6.0 mm, SNR 1.00, 6 mm section thickness. The scanning parameters of diffusion-weighted imaging (DWI) were TR 9000 ms, TE 66 ms, FOV 420 mm, matrix 80 × 148, voxel size 3.5 × 2.8 × 6.0 mm, SNR 1.00, 6 mm section thickness. Delay phase scanning was at 5, 10 and 20 min after GD-EOB-DTPA administration.

### Image segmentation

Arterial phase (AP), portal venous phase (PVP), delayed phase (DP) and hepatobiliary phase (HBP) images were transferred into the radiomics platform (Huiying Medical Technology Co., Ltd). Tumor MRI segmentation was performed manually by a junior radiologist with 2 years of professional experience, and reviewed by a senior radiologist who had 11 years of professional experience. The volumes of interest (VOIs) covered the whole tumor. Each phase of the lesion was mapped layer by layer manually. For well-defined lesions, we carefully delineate the edges of the lesions. For lesions with blurred boundaries, we comprehensively observed the imaging characteristics of each phase to determine the approximate boundary of the tumor and avoid overstepping the boundary when drawing.

### Feature extraction and machine learning

Quantitative image features were extracted from the tumors and divided into four subgroups: first-order statistical features, shape-based features, textural features, and higher order statistical features. The first-order statistical features provided the distribution of voxel intensities within the MR image via commonly used and basic metrics. The shape-based features provided how spherical, rounded, or elongated the tumor was. Textural features were computed using the gray level cooccurence matrix (GLCM), gray level run length matrix (GLRLM), and gray level size zone matrix (GLSZM). Higher order statistical features used Laplacian of Gaussian (LoG), wavelet, square, square root, logarithm, and exponential filters.

The predictive model was established by combining the extracted features through feature de-redundancy and dimensionality reduction, preconditioning, and machine learning-based classification. The least absolute shrinkage and selection operator (LASSO algorithm) was used to reduce dependency and redundancy. Preconditioning of the predictive model involved normalizing the data to ensure different features took on similar ranges of values. Classification was applied to identify DPHCC and CK7- and CK19-negative HCC on the basis of various features in multiple phases. A fivefold cross-validation was used to assess the performance of the classification models with receiver-operating curves (ROCs). Supervised learning used four classifiers, multi-layer perceptron (MLP), support vector machines (SVM), logistic regression (LR) and K-nearest neighbor (KNN), to identify a classifier that correctly classified new objects. Area under the curve (AUC), sensitivity, and specificity were significant indices for evaluating performance in differentiating DPHCC from CK7- and CK19-negative HCC on MRI.

### Prognostic analysis

All patients were followed up as outpatients or by telephone for 1–36 months after surgery. Tumor recurrence was identified using enhanced computed tomography (CT) or MRI. Data cut-off was April 30, 2019.

### Statistical analysis

Statistical analyses were performed using SPSS v22.0. Receiver operator characteristic (ROC) curves were constructed to demonstrate the predictive capability of the radiomic signature. Between-group comparisons were conducted with the Chi-squared test for categorical variables and the independent-sample *t* test for continuous variables. Significance was investigated with the Fisher’s exact probability method. Kaplan–Meier analyses were used for prognostic assessment. Significance was set at *P* < 0.05.

## Results

### Clinical features

The observation group included 50 patients (43 males and 7 females) with a postoperative pathological diagnosis of DPHCC and a mean age of 51.4 ± 11.2 years (range 33–79 years). Among the observation group, 41 patients were hepatitis B virus antigen (HBsAg) positive, 32 patients were alpha-fetoprotein (AFP) positive, and 17/22 patients were abnormal prothrombin (APT) positive. Based on histological grading, 2 tumors were Grade I, 31 tumors were Grade II, and 17 tumors were Grade III. 30 tumors were vascular endothelial growth factor (VEGF)-positive. The control group included 50 patients (43 males and 7 females) with CK7- and CK19-negtaive HCC and a mean age of 49.2 ± 11.9 years (range 28–70 years). Among the control group, 44 patients were HBsAg positive, 40 patients were AFP positive, and 5/6 patients were APT positive. Based on histological grading, 1 tumor was Grade I, 32 tumors were Grade II, and 17 tumors were Grade III. 37 tumors were VEGF positive. There were no statistically significant differences in clinical characteristics between the observation group and the control group (all *P* > 0.05) (Table 1).

**Table 1:** Patient clinical information

Information	DPHCC	HCC	*T* test
Gender (male/female)	43/7	43/7	*P* > 0.05
Age (years)	51.4 ± 11.2	49.2 ± 11.9	*P* > 0.05
HBsAg (+)	41/50	44/50	*P* > 0.05
AFP (+)	32/50	40/50	*P* > 0.05
APT (+)	17/22	5/6	*P* > 0.05
VEGF (+)	30/50	37/50	*P* > 0.05

*DPHCC* dual-phenotype hepatocellular carcinoma, *HCC* hepatocellular carcinoma, *AFP* alpha-fetoprotein, *APT* abnormal prothrombin, *VEGF* vascular endothelial growth factor

### Radiomics analysis

**Fig. 1:**
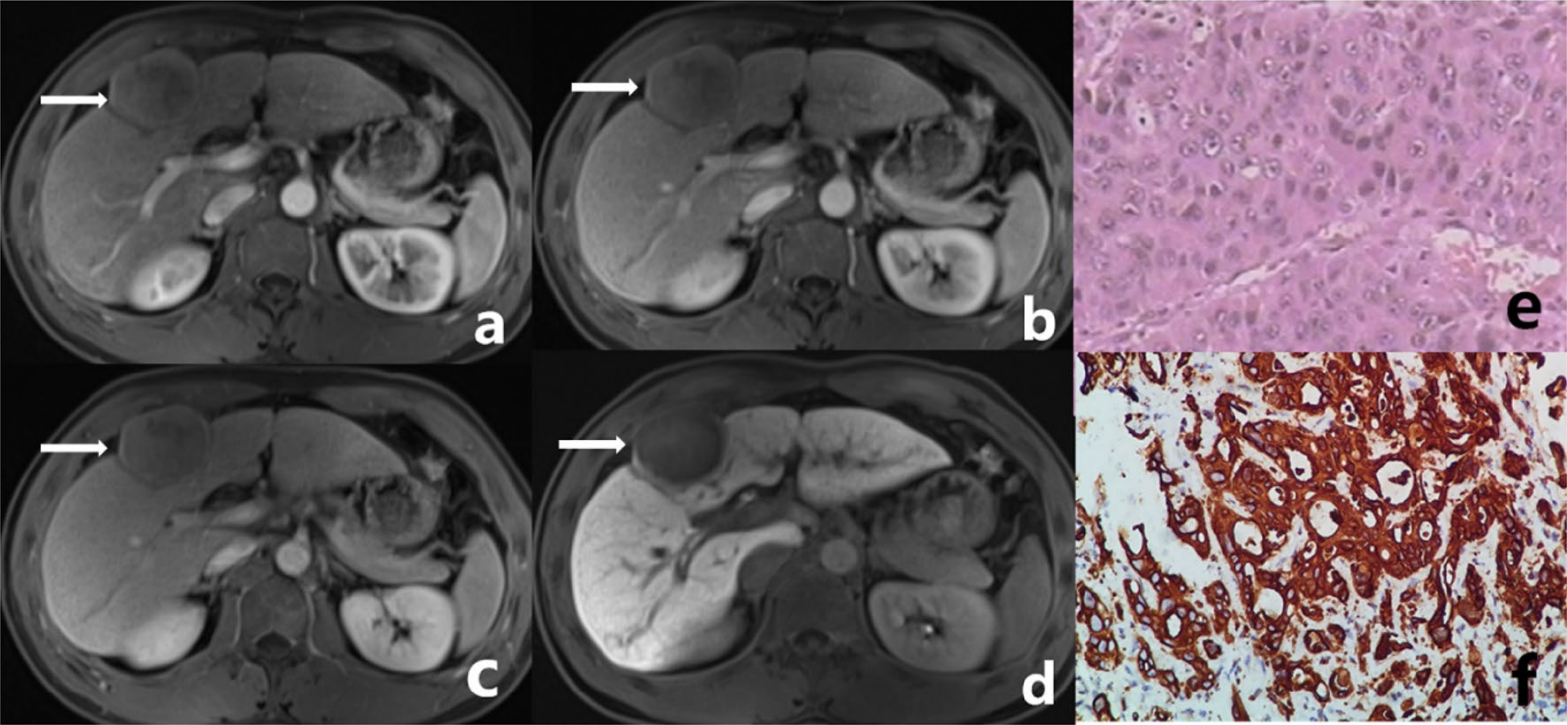
Male, 38 years old. A well-defined tumor in left inner lobe of liver (white arrow), which was confirmed DPHCC by postoperative pathology. **a** The tumor shows mild enhancement at arterial phase. **b**, **c** The degree of enhancement of the tumor is decreased in portal venous phase and delayed phase. **d** The tumor shows low signal in the hepatobiliary phase. **e** Microscopically, the tumor presents as trabecular type hepatocellular carcinoma in pathology. **f** CK19 (+++) in tumor immunohistochemistry

**Fig. 2:**
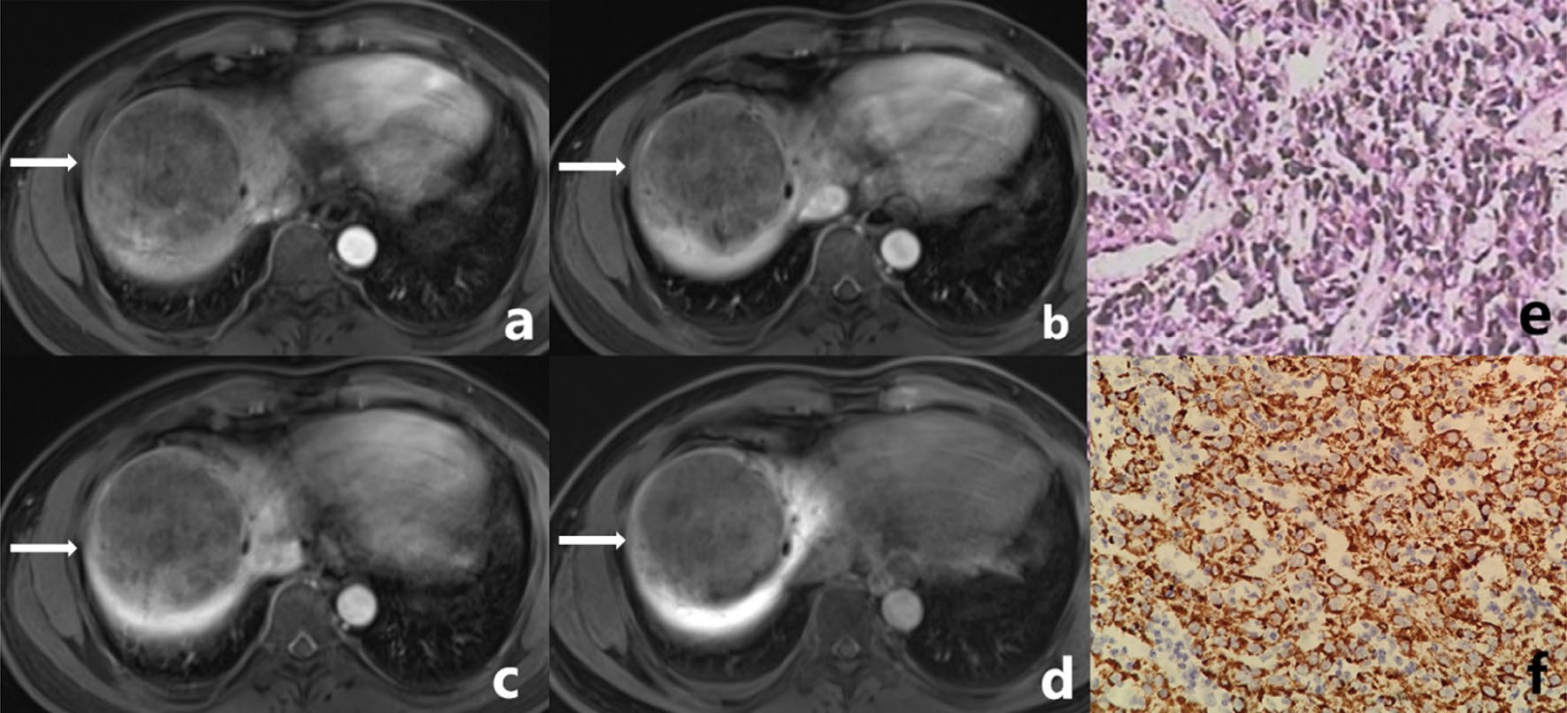
Male, 45 years old. A well-defined tumor in right lobe of liver (white arrow), which was confirmed CK7- and CK19-negative HCC by postoperative pathology. **a** The tumor shows mild inhomogeneous enhancement at arterial phase. **b**, **c** The degree of enhancement of the tumor is decreased in portal venous phase and delayed phase. **d** The tumor shows low signal in the hepatobiliary phase. **e** Microscopically, the tumor presents as trabecular type hepatocellular carcinoma in pathology. **f** Glypican-3 (++++) in tumor immunohistochemistry

The characteristics of preoperative MRI images and postoperative pathological morphology were similar between the observation group and the control group (Figs. 1, 2). It is difficult to differentiate and diagnose DPHCC by observation of human eyes before operation, so we use machines for feature analysis. A total of 1029 image features were extracted from the VOIs on AP, PVP, DP, and HBP images. A radiomics set was built after LASSO exported the optimal value of the LASSO tuning parameter (α = 1.0), features corresponding to the optimal α were derived, and coefficients were calculated (Table 2). There were seven features from the AP (two first-order statistical features, and five textural features [GLCM *n* = 1; GLRLM *n* = 1; GLSZM *n* = 3]), three features from the PVP (one first-order statistical feature, one shape-based feature, and one textural feature [GLSZM]), six features from the DP (two first-order statistical features, one shape-based feature, and three textural features [GLRLM *n* = 1; GLSZM *n* = 2]), and five features from the HBP (one first-order statistical feature, and four textural features [GLCM *n* = 3; GLSZM *n* = 1]). The maximum number of iterations was restricted to 5000.

**Table 2:** Extracted features and their coefficients (α = 1.0)

Image	Features	Coefficient values
AP	Logarithm_glcm_Idmn	− 0.00878758
	Wavelet-LHH_firstorder_Maximum	− 0.005271563
	Wavelet-HLL_glszm_ZoneEntropy	− 0.004053006
	Wavelet-HHH_firstorder_Skewness	0.008038893
	Wavelet-HHH_glszm_SmallAreaEmphasis	− 0.006191688
	Wavelet-HHH_glszm_ZoneEntropy	− 0.000869839
	Wavelet-HHH_glrlm_ShortRunLowGrayLevelEmphasis	0.012726913
PVP	Original_shape_Compactness2	0.010410787463874
	Wavelet-LHL_firstorder_Skewness	− 0.0154721553472323
	Wavelet-HHL_glszm_LowGrayLevelZoneEmphasis	0.0474535860866428
DP	Original_shape_Compactness2	0.0151357169669036
	Squareroot_glrlm_ShortRunLowGrayLevelEmphasis	0.000353944661747531
	Wavelet-LHL_firstorder_Skewness	− 0.0551567478294812
	Wavelet-LHL_glszm_SmallAreaEmphasis	− 0.00252715366295204
	Wavelet-LHH_glszm_LowGrayLevelZoneEmphasis	0.0147263733928842
	Wavelet-HHH_firstorder_Mean	0.0385977980471331
HBP	Original_glcm_Correlation	− 0.028752065
	Wavelet-HHL_firstorder_Mean	− 0.0237526
	Exponential_glcm_Imc2	− 0.005058007
	Wavelet-LHH_glcm_DifferenceAverage	− 0.004688264
	Wavelet-HLL_glszm_SmallAreaEmphasis	− 0.018554343

*AP* arterial phase, *PVP* portal venous phase, *DP* delayed phase, *HBP* hepatobiliary phase

**Fig. 3:**
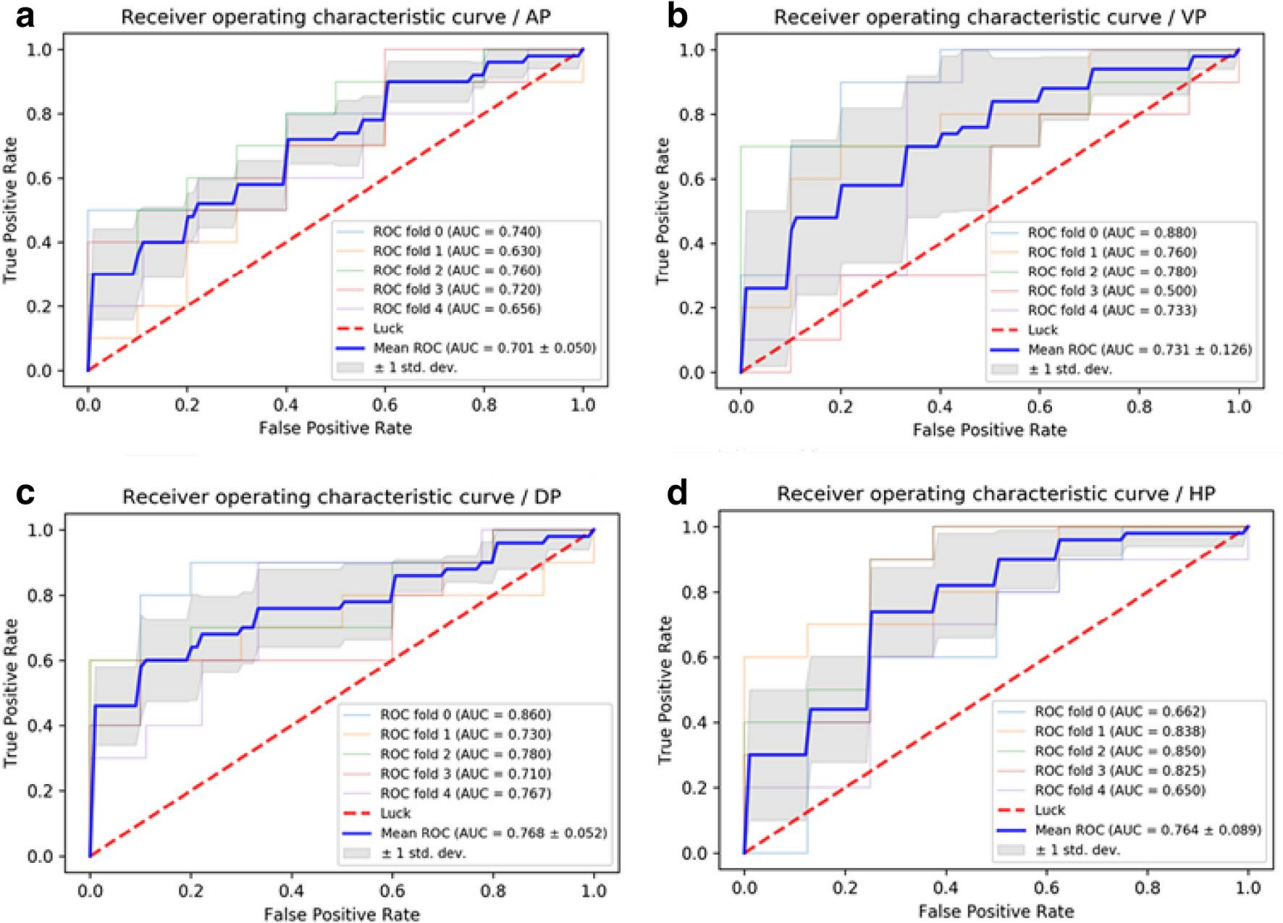
**a** ROC curves of LR methods to classification in arterial phase. **b** ROC curves of LR methods to classification in portal venous phase. **c** ROC curves of LR methods to classification in delayed phase. **d** ROC curves of LR methods to classification in hepatobiliary phase

**Fig. 4:**
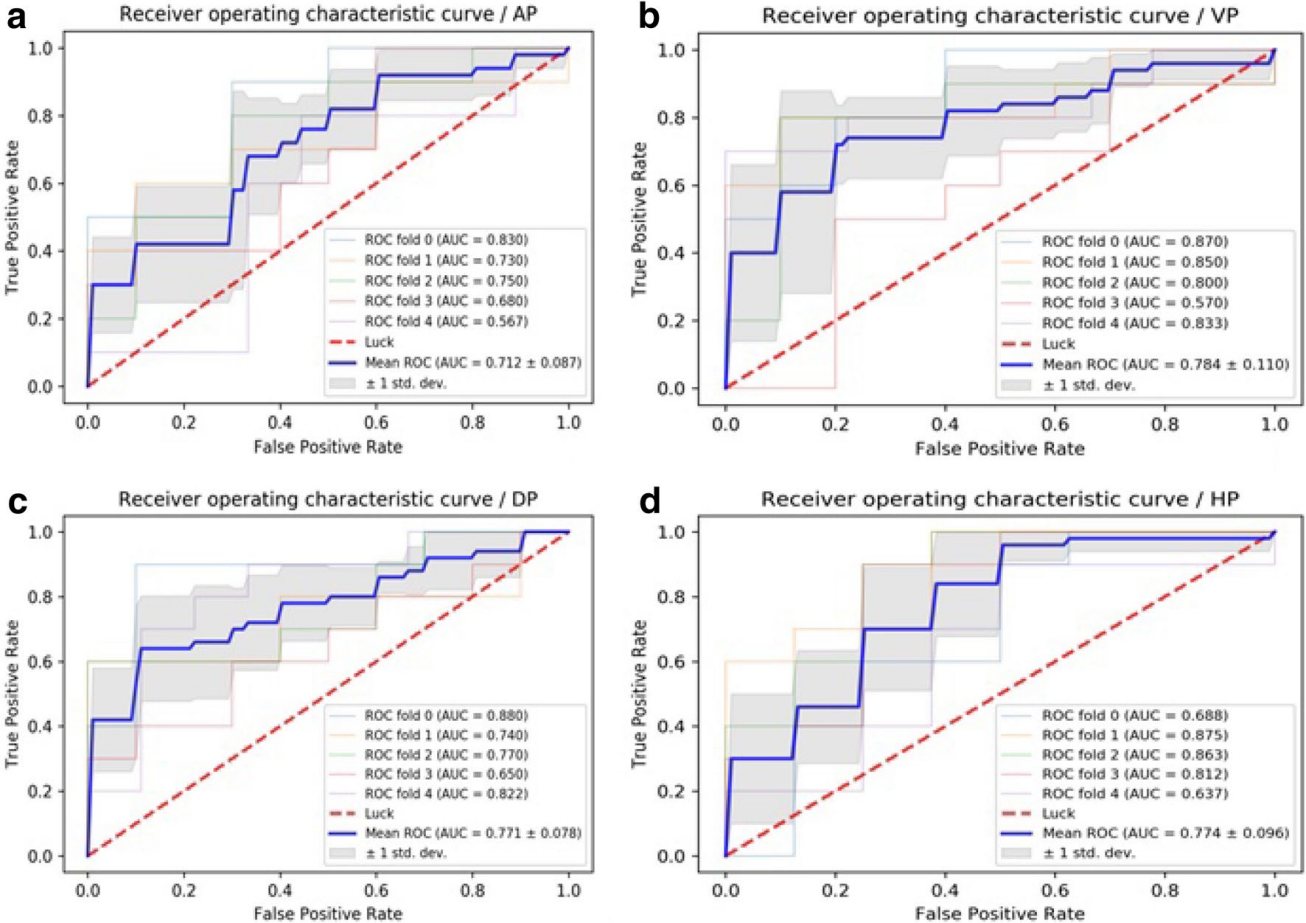
**a** ROC curves of MLP methods to classification in arterial phase. **b** ROC curves of MLP methods to classification in portal venous phase. **c** ROC curves of MLP methods to classification in delayed phase. **d** ROC curves of MLP methods to classification in hepatobiliary phase

Four classifiers were used to obtain the best model based on the feature set. ROC analysis revealed that LR and MLP showed the best performance (Figs. 3, 4) (Table 3).

**Table 3:** ROC analysis using fivefold cross-validation

Image	AUC of KNN	AUC of SVM	AUC of LR	AUC of MLP
AP	0.583 ± 0.104	0.302 ± 0.049	0.701 ± 0.050	0.712 ± 0.087
PVP	0.734 ± 0.145	0.617 ± 0.237	0.731 ± 0.126	0.784 ± 0.110
DP	0.677 ± 0.065	0.765 ± 0.047	0.768 ± 0.052	0.771 ± 0.078
HBP	0.675 ± 0.134	0.759 ± 0.091	0.764 ± 0.089	0.774 ± 0.096
Sensitivity				
AP	0.680	0.560	0.800	0.651
PVP	0.780	0.573	0.740	0.880
DP	0.840	0.874	0.893	0.938
HBP	0.650	0.775	0.800	0.625
Specificity				
AP	0.500	0.260	0.725	0.820
PVP	0.620	0.640	0.780	0.720
DP	0.480	0.700	0.700	0.640
HBP	0.620	0.760	0.720	0.920
Accuracy				
AP	0.596	0.324	0.696	0.737
PVP	0.696	0.606	0.766	0.798
DP	0.666	0.788	0.798	0.788
HBP	0.634	0.765	0.756	0.789
95% CI				
AP	0.597–0.769	0.219–0.388	0.631–0.797	0.632–0.795
PVP	0.807–0.915	0.633–0.803	0.639–0.807	0.932–0.998
DP	0.689–0.836	0.706–0.858	0.715–0.863	0.689–0.844
HBP	0.699–0.851	0.684–0.851	0.690–0.855	0.691–0.856

*AUC* area under receiver-operating characteristic curve, *AP* arterial phase, *PVP* portal venous phase, *DP* delayed phase, *HBP* hepatobiliary phase

### Prognosis

**Fig. 5:**
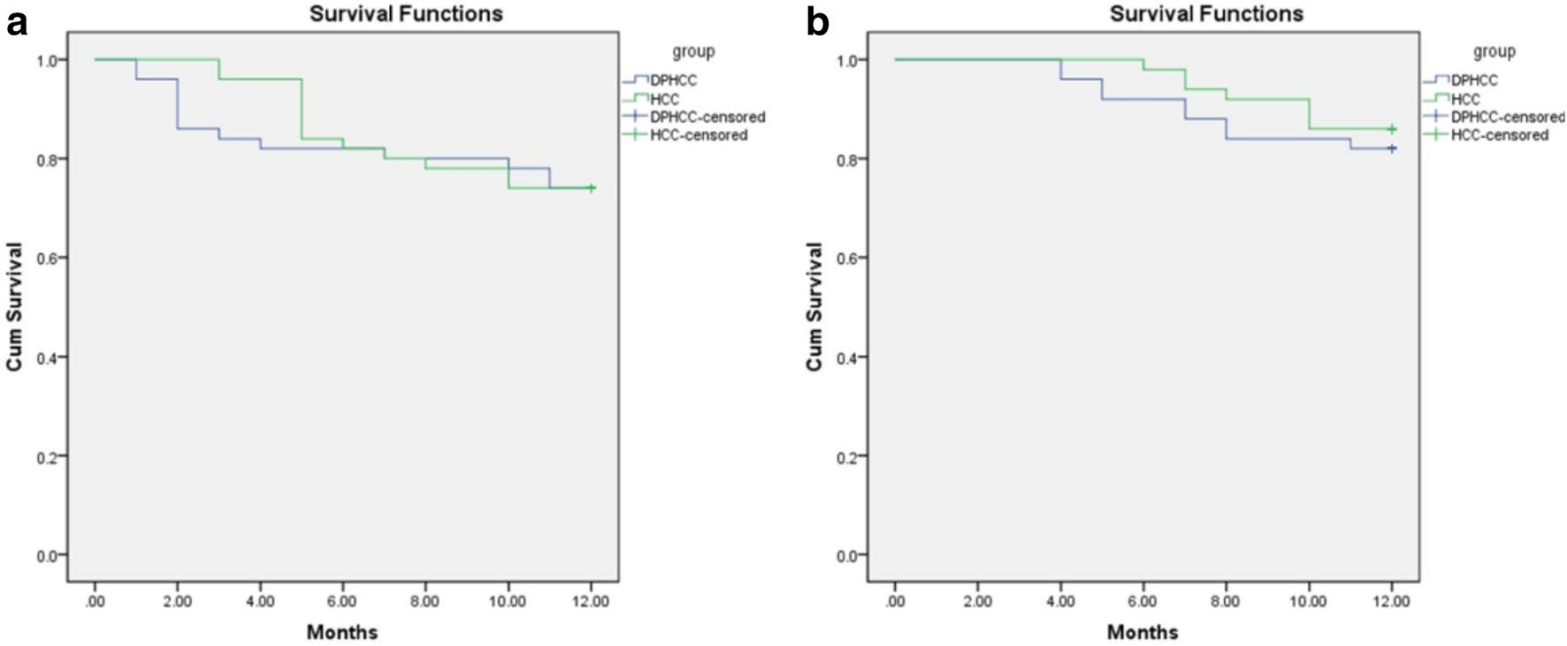
Kaplan–Meier survival analysis. **a** DFS of DPHCC and HCC. **b** OS of DPHCC and HCC

48/50 patients in the observation group and 49/50 patients in the control group were followed up until April 30, 2019; the contact information of the remaining patients changed, and they could not be reached. Forty-two patients in the observation group and 47 patients in the control group underwent surgery of hepatic resection before April 30, 2018. Patients who were followed-up for less than 1 year after surgery were excluded from the analyses. One-year no recurrence rate and overall survival rate of the patients in the observation group were 69% and 78%, respectively. The 1-year disease-free survival (DFS) and overall survival (OS) were 10.0 and 11.0 months, respectively. One-year no recurrence rate and overall survival rate of patients in the control group were 72% and 85%, respectively. The 1-year DFS and overall survival OS were 10.4 and 11.4 months, respectively. Kaplan–Meier survival analysis showed no statistical difference in DFS and OS between groups (*P* = 0.875 and 0.535) (Fig. 5).

## Discussion

### Clinical

Primary hepatocellular carcinoma can be classified as HCC, CC and combined HCC and cholangiocarcinoma (cHCC-CC) (Bosman et al. 2010). DPHCC is another subtype of HCC, in which tumor cells have typical morphological characteristics of HCC, but hepatocyte and cholangiocyte markers are strongly co-expressed within the same tumor cells (Cong et al. 2016; Li and Wang 2015). In the present study, there were no statistically significant differences in gender, age, HBsAg positivity, AFP positivity, histological grading and VGEF positivity in DPHCC or CK7- and CK19-negative HCC, but HBsAg positivity, AFP levels, and VGEF positivity were numerically lower in DPHCC, maybe suggesting that DPHCC is not as vascular as HCC. Further research is required to clearly characterize the pathophysiological characteristics of DHPCC.

### Radiomics

Liver cancer exhibits extraordinary morphological heterogeneity, (Li and Wang 2015) which is responsible for the inconsistent outcomes associated with anticancer therapies (Sequist et al. 2011). MRI is a non-invasive technique that allows evaluation of tumors (Lambin et al. 2012). Radiomics can be used to create signatures to characterize tumors based on features extracted from images. First-order features quantitatively delineate the distribution of voxel intensities within an image through commonly used and basic metrics. Shape-based features describe the three-dimensional size and shape of the VOI. Textural features are calculated with GLCM, which describes the arrangements of pairs of voxels with specific intensity values; GLRLM, which indicates the randomness in the distribution of running lengths in a region by quantifying gray level runs; and GLSZM, which indicates the randomness of gray levels in a region by quantifying gray level zones.

Four supervised learning classifiers were tested to create a classifier for DPHCC. LR and MLP showed the best performance having an AUC > 0.7, indicating good diagnostic capability. LR is used to address a regression or classification problem. LR assumes that data obey Bernoulli distribution, and uses gradient descent to solve parameters by maximizing the likelihood function, so as to achieve data dichotomy using a simple model with strong explanatory power. MLP is a feed-forward supervised neural network, which determines a non-linear mapping from an input vector to an output vector, parameterized by a set of network weights. In this study, LR and MLP were used for machine learning in each phase. The diagnostic capability of LR and MLP in the AP were inferior, possibly due to artifacts on Gd-EOB-DTPA-enhanced MRI (Tanimoto et al. 2012) affecting the extraction and calculation of textural-based features. The sensitivity, specificity and accuracy of LR and MLP varied with imaging phase, such that the best preoperative diagnostic power for DPHCC will likely be derived from a combination of different phases and classifiers (PVP, DP and HBP with LR and VP with MLP).

In a recent study, some scholars applied radiomics to achieve preoperative prediction of tumor-infiltrating CD8^+^ T cells in 142 patients with hepatocellular carcinoma by contrast-enhanced CT images (Liao et al. 2019). Chen et al. used radiomics to establish a clinical model of 207 patients with hepatocellular carcinoma with Gd-EOB-DTPA-enhanced MRI images, and predicted the immunoscore before treatment (Chen et al. 2019). CK7, CK19 are the immunohistochemical index of cholangiocellular carcinoma, which appear in DPHCC suggesting that HCC has the potential to differentiate into ICC. In this study, through the differentiation of DPHCC and CK7- and CK19-negative HCC used the radiomics of Gd-EOB-DTPA-enhanced MRI, we think that the features of radiomics may achieve the preoperative prediction of the immunohistochemical markers of cholangiocellular carcinoma (CK7, CK19).

### Prognosis

A previous study demonstrated high recurrence and mortality rates in patients with DPHCC (*n* = 155) and identified DPHCC as a tumor with high malignancy and poor prognosis (Cong et al. 2016). In the present study, based on 1 year of follow-up, there were no significant differences in DFS and OS in patients with DPHCC or CK7- and CK19-negative HCC. Although recurrence and mortality rates were numerically higher after 12 months of follow-up in patients with CK7- and CK19-negative HCC compared to those with DPHCC, DFS and OS were numerically lower in patients with DPHCC. This suggests that DPHCC is more likely to recur and cause death than HCC and that active postoperative management is required. Risk factors for recurrence and mortality in DPHCC include environmental and genetic susceptibilities, which require further investigation.

Recently, some scholars applied radiomics to predict the postoperative prognosis of HCC (Guo et al. 2019). Kim et al. used enhanced-MRI radiomics to prediction the early and late prognosis of 167 patients of HCC (Kim et al. 2019). In this study, radiomics was not used for prognostic analysis due to the small number of cases and short follow-up time. It remains to be further studied if we increase the sample size in the future.

## Conclusion

DPHCC is a novel subtype of HCC, which has similar imaging and clinical characteristics to HCC, but higher early recurrence and mortality rates. Radiomics features were extracted from Gd-EOB-DTPA-enhanced MR images. The best preoperative diagnostic power for DPHCC will likely be derived from a combination of different phases and classifiers. Further studies in a larger patient population are required to confirm these findings.

## Limitation

DPHCC was included in the pathological diagnostic guidelines since 2015, and it has gradually been recognized, but there were fewer patients with MRI examination, so the number of cases in this study was not large enough. More cases will be needed in the future to make the results more accurate.
